# Toxic epidermal necrosis associated with phenobarbitone: a case report and brief review of the literatures

**DOI:** 10.1186/s13223-021-00589-4

**Published:** 2021-09-08

**Authors:** Biniyam A. Ayele, Kemal Ali, Eliyas Mulatu

**Affiliations:** 1 Department of Neurology, School of Medicine, College of Health Sciences Addis Ababa University, Liberia Street, PO Box 6396, Addis Ababa, Ethiopia; 2 Department of Dermatovenereology, Addis Ababa University, Addis Ababa, Ethiopia

**Keywords:** Phenobarbitone, Toxic epidermal necrosis, Asthma, Hypersensitivity, Ethiopia

## Abstract

**Background:**

Toxic epidermal necrolysis (TEN)/Stevens–Johnson syndrome (SJS) is the spectrum of severe, acute, mucocutaneous, T-cell mediated delayed type IV hypersensitivity reaction and universally related to different drugs. Phenobarbitone is known to cause hypersensitivity reactions with benign pattern; ranging from a mild to moderate rashes but not life-threatening reactions such as TEN/SJS.

**Case report:**

We report a 14-year-old asthmatic male patient admitted to a local hospital for an acute exacerbation of asthma, after he presented with shortness of breath, cough, and fever. He was treated with bronchodilator and antibiotics. On subsequent days, the patient developed new onset generalized tonic clonic seizure in the hospital for which he was started on phenobarbitone of 100 mg twice daily. Two weeks after initiation of phenobarbitone, the patient developed extensive blistering skin eruptions; which subsequently exfoliated unevenly. Associated with the hypersensitivity skin reaction, the patient reported low grade fever, sore throat, and dysphagia. The exfoliation also involved oral and conjunctival mucosa; with estimated 65% body surface area involvement. The laboratory investigations were relevant for mild leucocytosis, prolonged prothrombin time, and reduced albumin. Phenobarbitone was discontinued and replaced with clonazepam; and the patient was managed with fluids replacement, IV antibiotics, twice daily wound care, analgesics, and naso gastric tube feeding. On subsequent days the patients’ clinical condition started improving; the skin lesion also started to heal and exfoliate in most of the affected skin surface areas, and the patient was discharged improved after ten days of intensive care unit.

**Conclusion:**

In summary, the present case describes, a 14-years-old young child with history of asthma and seizure disorder; and developed toxic epidermal necrosis following exposure to Phenobarbitone. This case also highlighted the better prognosis observed in pediatric population with TEN.

## Background

Toxic epidermal necrolysis (TEN)/Stevens–Johnson syndrome (SJS) is the spectrum of severe, acute, mucocutaneous, T-cell mediated delayed type IV hypersensitivity reaction; universally related to different drugs; which typically appears 1–3 weeks after the beginning of therapy. More than 100 medications have been implicated in this syndrome [1–4]**.** The incidence of TEN is 2 cases per million persons per year [5]. Phenobarbitone is known to cause hypersensitivity reactions with benign pattern; ranging from a mild to moderate rashes but not life-threatening reactions such as TEN/SJS. TEN is considerable associated with high morbidity and mortality; it is an exfoliative disease and results in full-thickness damage to the epidermis, characterized by a widespread bullae formation with epidermal necrosis and idiosyncratic of the skin and mucous membranes. TEN mainly occurs in adults and is often attributable to drug sensitivity and considered to be a severe form of Stevens–Johnson syndrome [1, 3, 5–8]. To the best of our knowledge, this is the first case of phenobarbitone-induced toxic epidermal necrosis in a young adolescent patient from the sub Saharan Africa.

## Case report

**Fig. 1:**
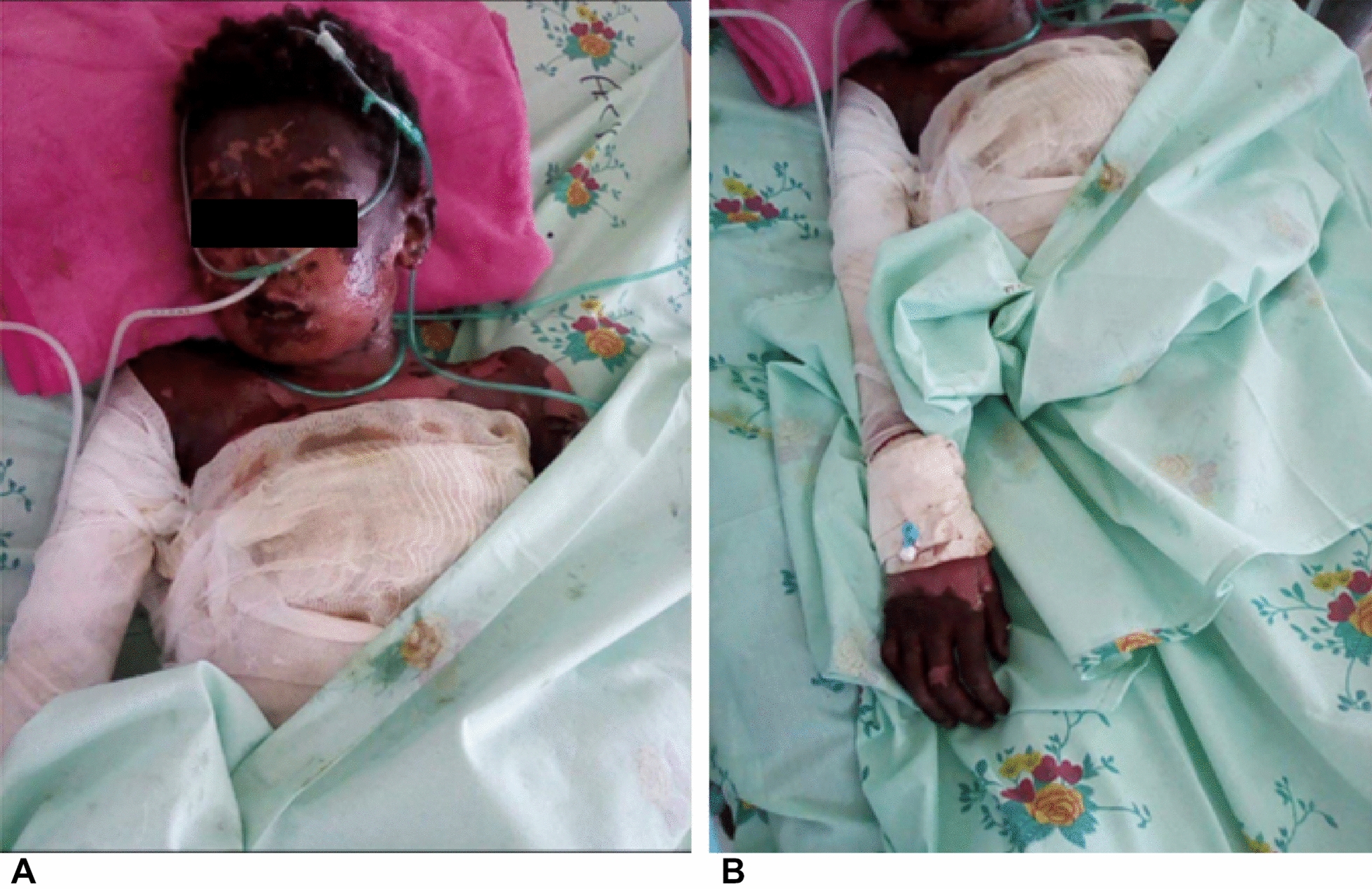
Showing ruptured blisters and extensive skin exfoliation involving face, oral cavity, chest, abdominal region (dressed) (**A**) right hand (**B**) (65% of total body surface area)

We report a 14-year-old asthmatic male patient admitted to a local hospital for an acute exacerbation of asthma, after he presented with shortness of breath, cough, and fever. He was treated with bronchodilator and antibiotics. On subsequent days, the patient developed new onset generalized tonic clonic seizure in the hospital for which he was started on Phenobarbitone of 100 mg twice daily. Two weeks after initiation of phenobarbitone, the patient developed extensive blistering skin eruptions; which later subsequently exfoliated unevenly. Associated with the hypersensitivity skin reaction, the patient reported low grade fever, sore throat, and dysphagia. The exfoliation also involved oral and conjunctival mucosa; with estimated 65% body surface area involvement. Up on presentation, blood pressure was 100/60 mmHg; pulse rate 100 beat per minutes; respiratory rate was 20 breath/minutes; temperature 38.3 °C; and oxygen saturation was 94% on atmospheric air. Oral examination shows, lesions involving his oral cavity and both lips. Ruptured blisters and extensive skin exfoliation was noted all over; involving approximately 65% of total body surface area (Fig. 1a, b). Laboratory investigations were unremarkable, except mild leucocytosis, prolonged prothrombin time, and reduced albumin; the rest of the laboratory investigations were summarized in the table below (Table 1). In the present case, skin biopsy was not performed, because, of the long appointment (1–3 months) for tissue histopathological tests at our hospital. The patient was admitted to medical intensive care unit (ICU) with consideration of phenobarbitone induced toxic epidermal necrosis, as the surface area affected by the exfoliating skin lesion was 65%; which fulfilled the criteria to diagnose TEN. Thus, Phenobarbitone was immediately discontinued and replaced with clonazepam; and in addition the patient was managed with fluids replacement, IV antibiotics, twice daily wound care, analgesics, and naso gastric tube feeding.

**Table 1:** List of patient’s laboratory investigations with normal reference value

		Normal reference values
White blood cells (WBC)	13, 400 (N 76%, L 11.8%)	5000–11,000 cells/mL
Hemoglobin (Hgb)	14.4 g/dL	14 – 16 g/dL
Mean corpuscular volume (MCV)	83.1 fL	80–99 fL
Platelets	302,000 cells/mL	150,000–350,000 cells/mL
Blood glucose	118 mg/dL	< 100 mg/dL
Creatinine	0.2 mg/dL	0.5–1.2 mg/dL
Blood urea nitrogen	11 mg/dL	5–18 mg/dL
ALT	16 IU/L	10–59 U/L
AST	10 IU/L	10–40 U/L
Alkaline phosphatase	70 IU/L	20–140 U/L
Sodium	137 mmol/L	135–146 mmol/L
Potassium	3.3 mmol/L	3.5–4.5 mmol/L
Chloride	97 mmol/L	96–106 mmol/L
Prothrombin time (PT)	18.1 s	12.0–14.0 s
Partial thromboplastin time (PTT)	41.6 s	20–35 s
INR	1.52	
Total bilirubin	0.4 mg/dL	0.2–1.3 mg/dL
Direct bilirubin	0.08 mg/dL	0.0–0.3 mg/dL
Albumin	2.2 g/dL	3.5–5.0 g/dL
HIV serology	Negative	

**Fig. 2:**
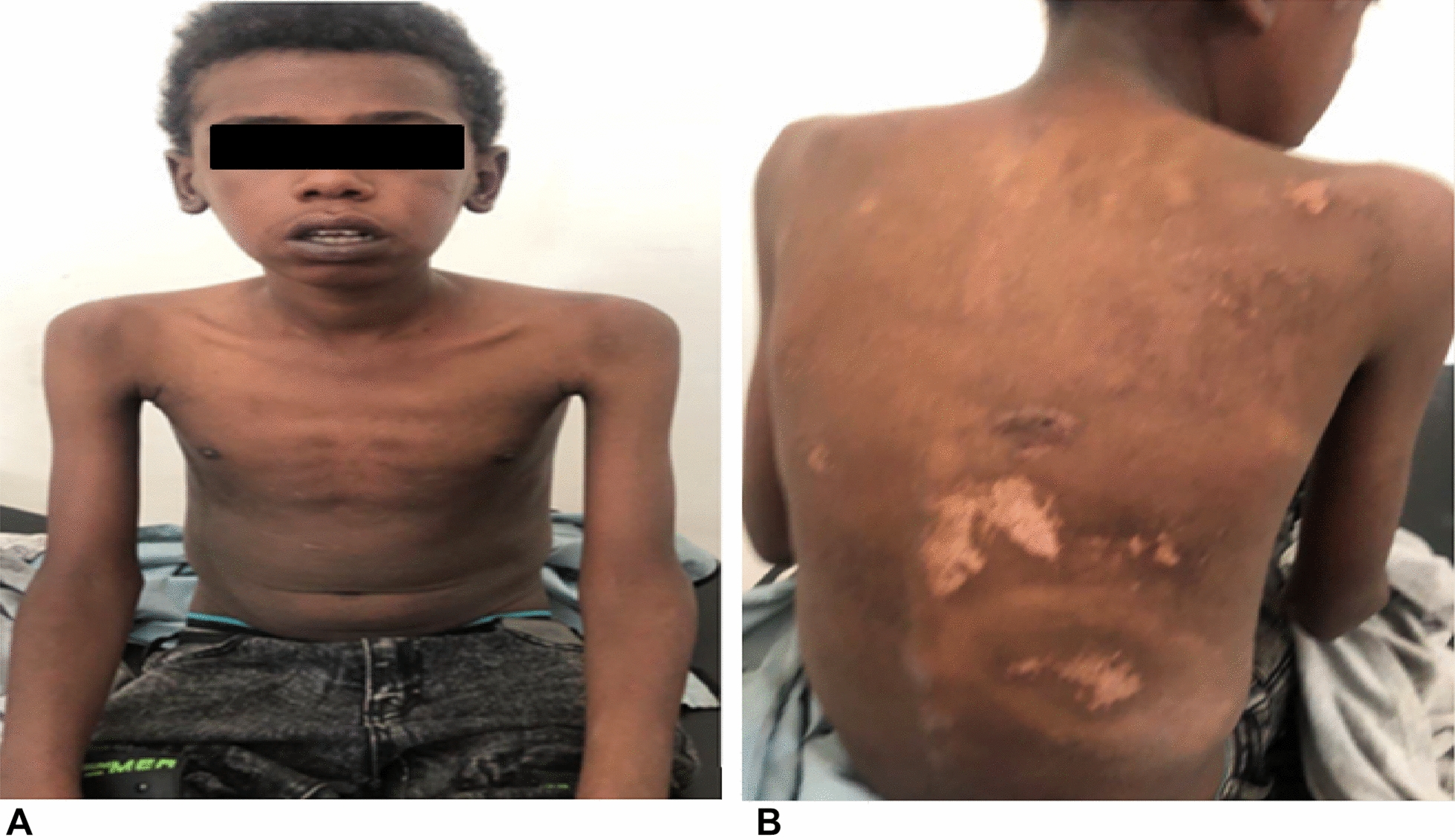
**A** Image showing complete resolution of the exfoliating skin lesion over the face, anterior chest, and hands. **B** Image showing patchy healing scars from the TEN leisons

In the present case, the prognosis was good with mortality rate of 12% based on SCORTEN scoring system [9] (Table 2). In this case, the only predictor of mortality was large surface area involvement of the blistering lesion (65%). According to SCORTEN, surface area involvement > 10% are considered poor prognostic factor (Table 2). On subsequent days in ICU, the patients’ clinical condition started improving; the skin lesion also started to heal and exfoliate in most of the affected skin surface areas. After 10 days of ICU management the patient was discharged home improvement and follow up appointment. On a subsequent follow up visit, the skin lesion on his face, anterior chest, and hands were fully recovered with scatted healing skin lesion on his back (Fig. 2a, b). He only reported pain on swallowing solid foods and he was advised on liquid and semisolid diet till he fully recovers.

**Table 2:** SCORTEN scoring of the patient [9]

Prognostic factors	Score	Mortality rate
Age > 40 years	0	Score 0 = 1–3% Score 2 = 12% Score 3 = 35% Score 4 = 58% Score 5 or more = 90%
Heart rate > 120 beats per minute	0	
Cancer or hematologic malignancy	0	
Involved body surface area > 10%	1	
Blood urea nitrogen level > 10 mmol/L (28 mg/dL)	0	
Serum bicarbonate level < 20 mmol/L (20 mEq/L)	0	
Blood glucose level > 14 mmol/L (252 mg/dL)	0

## Discussion and conclusion

The present case describes a child who presented with clinical features suggestive of TEN with large surface area involvement after exposure to phenobarbitone. The case also highlights the better prognosis observed in young patients with TEN. This case has comorbid asthma; asthmatics patients have a greater risk of developing adverse drug reactions (ADRs) and the majority of asthma patients are atopic; which will increase the risk of hypersensitive reactions [10, 11]. This is consistent with previous reports indicating risk factors of TEN/SJS including, history of allergy, advanced age, HIV infection, pre-existing liver disease, and chronic underlying diseases [12, 13]. The risk factors identified in the present case were history of allergy (e.g. asthma) and being on antiepileptic medications (e.g. epilepsy), which are one of the commonest types of medication with higher risk of developing TEN/SJS [3, 6, 14]. Thus, it’s important to follow a cautious approach when prescribing commonly known medications associated with TEN/SJS in patients having risk factors of TEN/SJS. The present case describes a case of TEN in a young boy. However, previous studies shows low prevalence of toxic epidermal necrosis in pediatric age group; furthermore, mortality rates in children with TEN are lower ranging from 0 to 7.5% compared to an overall mortality in adults which is ~30% [1, 8, 12, 13, 15]. Therefore, it’s vital to have high index of suspicion towards this severe adverse drug reactions characterized by a low incidence but high mortality, even in a young children.

Toxic epidermal necrosis is associated with drug exposure in up to 90% of the cases. These drugs includes: anticonvulsants, antibiotics, allopurinol, and non-steroidal anti-inflammatory [3, 4, 7, 16–18]. In the present case, the child was started on phenobarbitone, one of the common culprit drug associated with TEN [15, 18]. Likewise, other antiepileptic drugs (AED) were also incriminated with this deadly hypersensitivity skin reaction; few of these AEDs include: phenytoin, carbamazepine, oxcarbazepine, and lamotrigine [4, 16, 19, 20]. Therefore, clinicians should be aware of these drugs which are highly associated with TEN and should replace with drugs associated with lower skin hypersensitivity reactions. Skin and liver are the most affected organs by TEN [12, 13]. However, the present case had no clinical sign of liver injury, but had mild biochemical derangement suggestive of liver damage in the form of mild prolongation of prothrombin and partial thromboplastin time (Table 1). This finding further indicates the benign prognosis of TEN in pediatrics population.

Toxic epidermal necrosis is a multi-organ disease that not only affects the skin and mucous membranes but also several internal organs. Therefore, a multi-disciplinary approach is required. In a first step, immediate withdrawal of potentially causative drugs, ideally in the early stages of the disease, is mandatory to reduce fatality in SJS/TEN; in addition, supportive cares such as: fluid replacement, would care, and nutritional support is commended [1, 5, 8, 12, 15]. In the present case, the identified offending drug was Phenobarbitone, which we immediately discontinued. In addition, the child was given fluid replacement, intravenous antibiotics, and daily wound care; and discharged home improved.

Epilepsy is a common neurological disorder in sub-Saharan Africa; epilepsy is characterized as a chronic condition of recurrent unprovoked seizures [1, 15, 18, 21]. Antiepileptic drugs (AEDs) are vital in controlling the seizure attaché in epileptic patients. Thus, it’s important for the clinicians to be familiar with AEDs associated with highest incidences of TEN/SJS; these include: carbamazepine, lamotrigine, phenobarbital, phenytoin and valproic acid [1, 15, 18, 21]. Therefore, before prescribing these AEDs, it is important to ask history of drug allergy, atopic history, and family history of allergy; to avoid occurrence of delayed hypersensitivity reactions such as TEN.

In summary, the present case describes, a 14-years-old young child with history of allergy in a form of asthma and new onset seizure disorder; and developed toxic epidermal necrosis following exposure to phenobarbitone. This case also highlighted the better prognosis observed in pediatric population with TEN.

## Data Availability

All data sets on which the conclusions of the case report based, to be available as a medical record document and available from the corresponding author on reasonable request from the editors.
